# Aqua­(dicyanamido){μ-6,6′-dimeth­oxy-2,2′-[ethane-1,2-diylbis(nitrilo­methyl­idyne)]diphenolato}nickel(II)sodium

**DOI:** 10.1107/S160053680901438X

**Published:** 2009-04-22

**Authors:** Wei Wang, Yong-Miao Shen

**Affiliations:** a Yancheng Institute of Technology, School of Chemical and Biological Engineering, Yancheng 224003, People’s Republic of China; b Department of Chemistry, Shaoxing University, Shaoxing 312000, People’s Republic of China

## Abstract

The mol­ecule of the title compound, [NaNi(C_18_H_18_N_2_O_4_)(C_2_N_3_)(H_2_O)], is approximately planar, with a maximum deviation from the mol­ecular plane of 0.770 (5) Å. The coordination environment of the Ni^2+^ ion is distorted square-planar and it is N_2_O_2_ coordinated by the 6,6′-dimeth­oxy-2,2′-[ethane-1,2-diylbis(nitrilo­methyl­idyne)]diphenolate Schiff base ligand. The Na^+^ atom is chelated by the four O atoms of the Schiff base ligand, a water ligand and a dicyanamide anion. The structure displays inter­molecular O—H⋯N hydrogen bonding.

## Related literature

For chemical background, see: Ohba & Okawa (2000 ▶). For related structures, see: Correia *et al.* (2005 ▶); Costes *et al.*(2004 ▶).
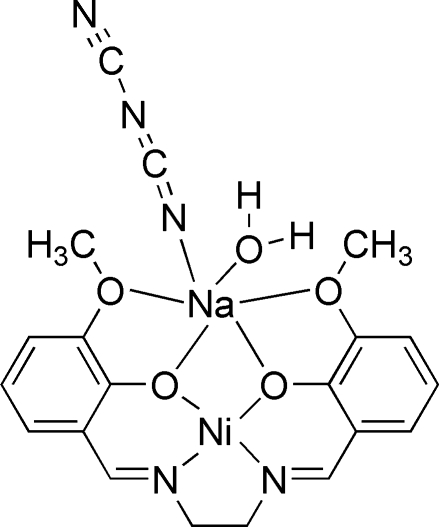


## Experimental

### 

#### Crystal data

[NaNi(C_18_H_18_N_2_O_4_)(C_2_N_3_)(H_2_O)]
*M* *_r_* = 492.11
Monoclinic, 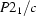

*a* = 7.4654 (14) Å
*b* = 22.745 (4) Å
*c* = 13.177 (3) Å
β = 101.282 (4)°
*V* = 2194.2 (8) Å^3^
*Z* = 4
Mo *K*α radiation
μ = 0.95 mm^−1^
*T* = 293 K
0.14 × 0.13 × 0.11 mm

#### Data collection

Bruker SMART CCD area-detector diffractometer
Absorption correction: multi-scan (*SADABS*; Sheldrick, 2003 ▶) *T*_min_ = 0.879, *T*_max_ = 0.903
10817 measured reflections
3864 independent reflections
2815 reflections with *I* > 2σ(*I*)
*R*_int_ = 0.032

#### Refinement

*R*[*F* ^2^ > 2σ(*F* ^2^)] = 0.039
*wR*(*F* ^2^) = 0.099
*S* = 1.02
3864 reflections
291 parameters
54 restraints
H-atom parameters constrained
Δρ_max_ = 0.33 e Å^−3^
Δρ_min_ = −0.35 e Å^−3^

### 

Data collection: *APEX2* (Bruker, 2004 ▶); cell refinement: *SAINT-Plus* (Bruker, 2001 ▶); data reduction: *SAINT-Plus*; program(s) used to solve structure: *SHELXS97* (Sheldrick, 2008 ▶); program(s) used to refine structure: *SHELXL97* (Sheldrick, 2008 ▶); molecular graphics: *SHELXTL* (Sheldrick, 2008 ▶); software used to prepare material for publication: *SHELXTL*.

## Supplementary Material

Crystal structure: contains datablocks I, global. DOI: 10.1107/S160053680901438X/hg2500sup1.cif

Structure factors: contains datablocks I. DOI: 10.1107/S160053680901438X/hg2500Isup2.hkl

Additional supplementary materials:  crystallographic information; 3D view; checkCIF report

## Figures and Tables

**Table 1:** Hydrogen-bond geometry (Å, °)

*D*—H⋯*A*	*D*—H	H⋯*A*	*D*⋯*A*	*D*—H⋯*A*
O5—H5*A*⋯N3^i^	0.82	2.14	2.960 (4)	175
O5—H5*B*⋯N4^ii^	0.82	2.03	2.852 (4)	177

Symmetry codes: (i) 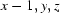; (ii) 
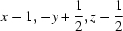
.

**Fig. 1.**
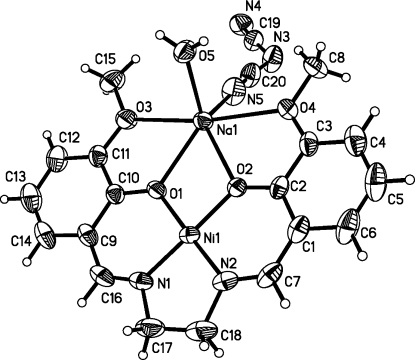
The independent molecules of the title compound, showing 30% probability displacement ellipsoids and the atom-numbering scheme.

## supplementary crystallographic information

### Comment

The dicyanamide ligand N(CN)_2_, has attracted continuous attention in the past four years for the buildup of interesting extended architectures. Its versatile coordination behavior and its ability to organize solids into polymeric structures with a rich diversity of magnetic properties have attracted interest toward this research area (Ohba *et al.*, 2000). *N*,*N*-disalicylideneethylenediamine type Schiff bases ligands present versatile steric, electronic and lipophilic properties (Correia *et al.* 2005). We report here the synthesis and crystal structure of the title compound. The molecular structure is shown in Fig. 1. The values of the geometric parameters in this compound are normal (Costes *et al.*, 2004). Ni^II^ and Na^I^ are connected *via* two bridging O atoms of the ligand. The six-coordinate Na atom adopts a distorted octahedral coordination geometry while the four-coordinate Ni gives a planar coordination geometry.

### Experimental

A mixture of 6,6'-dimethoxy-2,2'-(ethane-1,2-diyldiiminodimethylene)diphenol (1 mmol) and nickel chloride (1 mmol) in methanol (15 ml) was stirred for 30 min and sodium dicyanamide (1 mmol) was added, stirred for another 15 min and then filtered. The resulting clear orange solution was vapor at room temperature for 7 d, after which large orange block-shaped crystals of the title complex suitable for X-ray diffraction analysis were obtained.

### Refinement

The H atoms were fixed geometrically and were treated as riding on their parent C atoms, with C—H distances in the range of 0.93–0.97 Å and with *U*_iso_(H) = 1.2*U*_eq_(parent atom), or *U*_iso_(H) = 1.5*U*_eq_(C_methyl_).

### Crystal data

**Table:**

[NaNi(C_18_H_18_N_2_O_4_)(C_2_N_3_)(H_2_O)]	*F*(000) = 1016
*M**_r_* = 492.11	*D*_x_ = 1.490 Mg m^−^^3^
Monoclinic, *P*2_1_/*c*	Mo *K*α radiation, λ = 0.71073 Å
Hall symbol: -P 2ybc	Cell parameters from 3120 reflections
*a* = 7.4654 (14) Å	θ = 2.5–24.6°
*b* = 22.745 (4) Å	µ = 0.95 mm^−^^1^
*c* = 13.177 (3) Å	*T* = 293 K
β = 101.282 (4)°	Block, orange
*V* = 2194.2 (8) Å^3^	0.14 × 0.13 × 0.11 mm
*Z* = 4	

### Data collection

**Table:**

Bruker SMART CCD area-detector diffractometer	3864 independent reflections
Radiation source: fine-focus sealed tube	2815 reflections with *I* > 2σ(*I*)
graphite	*R*_int_ = 0.032
φ and ω scans	θ_max_ = 25.0°, θ_min_ = 1.8°
Absorption correction: multi-scan (*SADABS*; Sheldrick, 2003)	*h* = −8→8
*T*_min_ = 0.879, *T*_max_ = 0.903	*k* = −26→27
10817 measured reflections	*l* = −15→14

### Refinement

**Table:**

Refinement on *F*^2^	Primary atom site location: structure-invariant direct methods
Least-squares matrix: full	Secondary atom site location: difference Fourier map
*R*[*F*^2^ > 2σ(*F*^2^)] = 0.039	Hydrogen site location: inferred from neighbouring sites
*wR*(*F*^2^) = 0.099	H-atom parameters constrained
*S* = 1.02	*w* = 1/[σ^2^(*F*_o_^2^) + (0.0455*P*)^2^ + 0.4335*P*] where *P* = (*F*_o_^2^ + 2*F*_c_^2^)/3
3864 reflections	(Δ/σ)_max_ = 0.001
291 parameters	Δρ_max_ = 0.33 e Å^−^^3^
54 restraints	Δρ_min_ = −0.35 e Å^−^^3^

### Special details

**Table:**

Geometry. All e.s.d.'s (except the e.s.d. in the dihedral angle between two l.s. planes) are estimated using the full covariance matrix. The cell e.s.d.'s are taken into account individually in the estimation of e.s.d.'s in distances, angles and torsion angles; correlations between e.s.d.'s in cell parameters are only used when they are defined by crystal symmetry. An approximate (isotropic) treatment of cell e.s.d.'s is used for estimating e.s.d.'s involving l.s. planes.
Refinement. Refinement of *F*^2^ against ALL reflections. The weighted *R*-factor *wR* and goodness of fit *S* are based on *F*^2^, conventional *R*-factors *R* are based on *F*, with *F* set to zero for negative *F*^2^. The threshold expression of *F*^2^ > σ(*F*^2^) is used only for calculating *R*-factors(gt) *etc*. and is not relevant to the choice of reflections for refinement. *R*-factors based on *F*^2^ are statistically about twice as large as those based on *F*, and *R*- factors based on ALL data will be even larger.

### Fractional atomic coordinates and isotropic or equivalent isotropic displacement parameters (Å^2^)

**Table:**

	*x*	*y*	*z*	*U*_iso_*/*U*_eq_	
Ni1	0.24893 (5)	0.506776 (16)	0.06029 (3)	0.04684 (15)	
Na1	0.36614 (16)	0.38280 (5)	0.20328 (9)	0.0527 (3)	
O1	0.2723 (3)	0.48215 (8)	0.19509 (16)	0.0545 (5)	
O2	0.3315 (3)	0.43203 (8)	0.03988 (15)	0.0497 (5)	
O3	0.3154 (4)	0.42650 (10)	0.36882 (17)	0.0732 (7)	
O4	0.4284 (3)	0.32315 (9)	0.04958 (19)	0.0655 (6)	
O5	0.1624 (3)	0.30933 (10)	0.21624 (17)	0.0731 (7)	
H5A	0.1005	0.3032	0.2601	0.088*	
H5B	0.1305	0.2871	0.1665	0.088*	
N1	0.1665 (3)	0.58076 (11)	0.0844 (2)	0.0584 (7)	
N2	0.2232 (3)	0.52892 (13)	−0.0760 (2)	0.0589 (7)	
N3	0.9189 (4)	0.28821 (17)	0.3648 (3)	0.0917 (9)	
N4	1.0360 (5)	0.26666 (15)	0.5450 (3)	0.0888 (10)	
N5	0.6431 (5)	0.34246 (17)	0.2954 (3)	0.0934 (9)	
C1	0.3049 (4)	0.43665 (18)	−0.1445 (3)	0.0652 (10)	
C2	0.3415 (4)	0.40716 (15)	−0.0485 (2)	0.0511 (8)	
C3	0.3908 (4)	0.34689 (15)	−0.0474 (3)	0.0601 (9)	
C4	0.4007 (6)	0.3183 (2)	−0.1377 (4)	0.0885 (13)	
H4	0.4321	0.2787	−0.1361	0.106*	
C5	0.3642 (7)	0.3480 (3)	−0.2315 (4)	0.1134 (17)	
H5	0.3708	0.3281	−0.2923	0.136*	
C6	0.3189 (6)	0.4060 (3)	−0.2353 (3)	0.0998 (15)	
H6	0.2969	0.4256	−0.2985	0.120*	
C7	0.2509 (5)	0.49684 (19)	−0.1515 (3)	0.0694 (11)	
H7	0.2342	0.5144	−0.2164	0.083*	
C8	0.4977 (6)	0.26446 (15)	0.0610 (3)	0.0903 (13)	
H8A	0.4044	0.2375	0.0292	0.135*	
H8B	0.5337	0.2553	0.1332	0.135*	
H8C	0.6014	0.2611	0.0283	0.135*	
C9	0.1804 (5)	0.57147 (14)	0.2674 (3)	0.0631 (9)	
C10	0.2389 (4)	0.51231 (13)	0.2744 (3)	0.0524 (8)	
C11	0.2599 (5)	0.48361 (15)	0.3708 (3)	0.0615 (9)	
C12	0.2255 (6)	0.5127 (2)	0.4565 (3)	0.0842 (12)	
H12	0.2398	0.4933	0.5196	0.101*	
C13	0.1694 (7)	0.5711 (2)	0.4492 (4)	0.0982 (14)	
H13	0.1469	0.5905	0.5076	0.118*	
C14	0.1473 (6)	0.59969 (18)	0.3580 (4)	0.0854 (12)	
H14	0.1095	0.6387	0.3543	0.102*	
C15	0.3392 (6)	0.39343 (19)	0.4619 (3)	0.0919 (13)	
H15A	0.4253	0.4130	0.5147	0.138*	
H15B	0.3841	0.3550	0.4502	0.138*	
H15C	0.2241	0.3900	0.4836	0.138*	
C17	0.1111 (6)	0.61685 (18)	−0.0096 (4)	0.0889 (13)	
H16A	−0.0209	0.6203	−0.0257	0.107*	
H16B	0.1619	0.6560	0.0028	0.107*	
C18	0.1737 (7)	0.59090 (18)	−0.0957 (4)	0.0952 (14)	
H17A	0.2791	0.6124	−0.1086	0.114*	
H17B	0.0782	0.5938	−0.1571	0.114*	
C19	0.9741 (5)	0.27798 (17)	0.4624 (3)	0.0681 (9)	
C20	0.7695 (6)	0.31745 (18)	0.3323 (3)	0.0762 (9)	
C16	0.1480 (5)	0.60153 (15)	0.1717 (3)	0.0691 (10)	
H20	0.1091	0.6404	0.1724	0.083*	

### Atomic displacement parameters (Å^2^)

**Table:**

	*U*^11^	*U*^22^	*U*^33^	*U*^12^	*U*^13^	*U*^23^
Ni1	0.0435 (2)	0.0440 (2)	0.0519 (3)	−0.00269 (17)	0.00649 (17)	0.00924 (18)
Na1	0.0576 (7)	0.0475 (7)	0.0521 (7)	0.0048 (6)	0.0090 (6)	0.0046 (5)
O1	0.0737 (15)	0.0407 (11)	0.0502 (13)	0.0055 (10)	0.0146 (11)	−0.0009 (10)
O2	0.0576 (13)	0.0493 (12)	0.0426 (12)	0.0010 (10)	0.0109 (10)	0.0014 (9)
O3	0.115 (2)	0.0601 (15)	0.0482 (14)	0.0084 (14)	0.0235 (13)	0.0051 (11)
O4	0.0800 (16)	0.0474 (13)	0.0746 (17)	−0.0003 (11)	0.0284 (13)	−0.0082 (12)
O5	0.0885 (17)	0.0713 (15)	0.0650 (16)	−0.0195 (13)	0.0290 (13)	−0.0105 (12)
N1	0.0475 (16)	0.0432 (15)	0.081 (2)	−0.0028 (12)	0.0046 (14)	0.0120 (15)
N2	0.0461 (16)	0.0653 (18)	0.0625 (19)	−0.0061 (13)	0.0036 (14)	0.0215 (15)
N3	0.0765 (19)	0.131 (2)	0.0687 (18)	0.0310 (17)	0.0164 (15)	0.0124 (18)
N4	0.099 (2)	0.085 (2)	0.076 (2)	0.0171 (19)	0.0042 (18)	0.0172 (18)
N5	0.0782 (19)	0.125 (2)	0.0725 (19)	0.0268 (18)	0.0037 (16)	0.0108 (17)
C1	0.046 (2)	0.100 (3)	0.049 (2)	−0.0013 (19)	0.0089 (15)	−0.001 (2)
C2	0.0405 (17)	0.070 (2)	0.0432 (19)	−0.0088 (16)	0.0099 (14)	−0.0071 (16)
C3	0.056 (2)	0.067 (2)	0.060 (2)	−0.0097 (17)	0.0196 (17)	−0.0206 (19)
C4	0.088 (3)	0.097 (3)	0.083 (3)	−0.003 (2)	0.023 (2)	−0.034 (3)
C5	0.119 (4)	0.159 (5)	0.066 (3)	0.015 (4)	0.027 (3)	−0.038 (3)
C6	0.097 (3)	0.160 (5)	0.044 (2)	0.008 (3)	0.018 (2)	−0.009 (3)
C7	0.051 (2)	0.108 (3)	0.048 (2)	−0.007 (2)	0.0077 (16)	0.021 (2)
C8	0.111 (3)	0.045 (2)	0.124 (4)	0.000 (2)	0.045 (3)	−0.009 (2)
C9	0.057 (2)	0.052 (2)	0.079 (3)	0.0015 (16)	0.0093 (18)	−0.0180 (19)
C10	0.0513 (19)	0.0493 (18)	0.057 (2)	−0.0025 (15)	0.0122 (15)	−0.0081 (16)
C11	0.066 (2)	0.065 (2)	0.056 (2)	−0.0009 (18)	0.0165 (17)	−0.0114 (18)
C12	0.097 (3)	0.100 (3)	0.057 (3)	0.000 (3)	0.019 (2)	−0.018 (2)
C13	0.111 (4)	0.099 (4)	0.088 (4)	0.006 (3)	0.027 (3)	−0.043 (3)
C14	0.083 (3)	0.071 (3)	0.102 (3)	0.015 (2)	0.016 (2)	−0.033 (3)
C15	0.124 (4)	0.096 (3)	0.058 (2)	0.001 (3)	0.025 (2)	0.022 (2)
C17	0.079 (3)	0.075 (3)	0.113 (4)	0.017 (2)	0.018 (3)	0.047 (3)
C18	0.108 (3)	0.080 (3)	0.094 (3)	0.005 (3)	0.011 (3)	0.041 (3)
C19	0.0625 (19)	0.080 (2)	0.0614 (18)	0.0125 (16)	0.0121 (16)	0.0107 (18)
C20	0.068 (2)	0.105 (2)	0.0550 (18)	0.0168 (19)	0.0113 (16)	0.0098 (18)
C16	0.061 (2)	0.0391 (18)	0.102 (3)	0.0030 (16)	0.004 (2)	−0.006 (2)

### Geometric parameters (Å, °)

**Table:**

Ni1—O1	1.838 (2)	C3—C4	1.371 (5)
Ni1—N2	1.839 (3)	C4—C5	1.387 (6)
Ni1—N1	1.840 (3)	C4—H4	0.9300
Ni1—O2	1.8457 (19)	C5—C6	1.361 (7)
Na1—O5	2.288 (2)	C5—H5	0.9300
Na1—O1	2.362 (2)	C6—H6	0.9300
Na1—N5	2.368 (4)	C7—H7	0.9300
Na1—O2	2.395 (2)	C8—H8A	0.9600
Na1—O3	2.492 (3)	C8—H8B	0.9600
Na1—O4	2.555 (2)	C8—H8C	0.9600
O1—C10	1.314 (4)	C9—C10	1.412 (4)
O2—C2	1.310 (3)	C9—C16	1.413 (5)
O3—C11	1.365 (4)	C9—C14	1.419 (5)
O3—C15	1.420 (4)	C10—C11	1.410 (5)
O4—C3	1.365 (4)	C11—C12	1.375 (5)
O4—C8	1.429 (4)	C12—C13	1.389 (6)
O5—H5A	0.8200	C12—H12	0.9300
O5—H5B	0.8246	C13—C14	1.349 (6)
N1—C16	1.276 (4)	C13—H13	0.9300
N1—C17	1.476 (4)	C14—H14	0.9300
N2—C7	1.282 (4)	C15—H15A	0.9600
N2—C18	1.468 (5)	C15—H15B	0.9600
N3—C19	1.292 (5)	C15—H15C	0.9600
N3—C20	1.297 (5)	C17—C18	1.436 (6)
N4—C19	1.127 (4)	C17—H16A	0.9700
N5—C20	1.127 (4)	C17—H16B	0.9700
C1—C6	1.405 (5)	C18—H17A	0.9700
C1—C2	1.411 (5)	C18—H17B	0.9700
C1—C7	1.425 (5)	C16—H20	0.9300
C2—C3	1.419 (5)		
			
O1—Ni1—N2	178.09 (11)	C6—C5—C4	120.6 (4)
O1—Ni1—N1	94.79 (11)	C6—C5—H5	119.7
N2—Ni1—N1	86.76 (14)	C4—C5—H5	119.7
O1—Ni1—O2	83.62 (9)	C5—C6—C1	120.5 (4)
N2—Ni1—O2	94.82 (11)	C5—C6—H6	119.7
N1—Ni1—O2	178.41 (11)	C1—C6—H6	119.7
O5—Na1—O1	120.42 (9)	N2—C7—C1	125.7 (3)
O5—Na1—N5	101.85 (12)	N2—C7—H7	117.1
O1—Na1—N5	127.99 (12)	C1—C7—H7	117.1
O5—Na1—O2	116.86 (9)	O4—C8—H8A	109.5
O1—Na1—O2	62.15 (7)	O4—C8—H8B	109.5
N5—Na1—O2	124.88 (11)	H8A—C8—H8B	109.5
O5—Na1—O3	90.51 (9)	O4—C8—H8C	109.5
O1—Na1—O3	64.15 (8)	H8A—C8—H8C	109.5
N5—Na1—O3	88.43 (11)	H8B—C8—H8C	109.5
O2—Na1—O3	126.30 (8)	C10—C9—C16	121.1 (3)
O5—Na1—O4	84.15 (8)	C10—C9—C14	118.6 (4)
O1—Na1—O4	124.77 (9)	C16—C9—C14	120.2 (4)
N5—Na1—O4	85.68 (11)	O1—C10—C11	118.0 (3)
O2—Na1—O4	62.63 (7)	O1—C10—C9	123.4 (3)
O3—Na1—O4	171.07 (9)	C11—C10—C9	118.6 (3)
C10—O1—Ni1	127.8 (2)	O3—C11—C12	125.5 (4)
C10—O1—Na1	124.3 (2)	O3—C11—C10	113.8 (3)
Ni1—O1—Na1	107.87 (9)	C12—C11—C10	120.7 (4)
C2—O2—Ni1	127.3 (2)	C11—C12—C13	120.4 (4)
C2—O2—Na1	125.61 (19)	C11—C12—H12	119.8
Ni1—O2—Na1	106.26 (9)	C13—C12—H12	119.8
C11—O3—C15	118.3 (3)	C14—C13—C12	120.3 (4)
C11—O3—Na1	119.68 (19)	C14—C13—H13	119.8
C15—O3—Na1	122.0 (2)	C12—C13—H13	119.8
C3—O4—C8	118.2 (3)	C13—C14—C9	121.3 (4)
C3—O4—Na1	119.73 (19)	C13—C14—H14	119.3
C8—O4—Na1	122.1 (2)	C9—C14—H14	119.3
Na1—O5—H5A	130.6	O3—C15—H15A	109.5
Na1—O5—H5B	118.8	O3—C15—H15B	109.5
H5A—O5—H5B	110.0	H15A—C15—H15B	109.5
C16—N1—C17	119.2 (3)	O3—C15—H15C	109.5
C16—N1—Ni1	126.4 (2)	H15A—C15—H15C	109.5
C17—N1—Ni1	114.3 (3)	H15B—C15—H15C	109.5
C7—N2—C18	118.9 (3)	C18—C17—N1	110.7 (3)
C7—N2—Ni1	126.8 (3)	C18—C17—H16A	109.5
C18—N2—Ni1	114.2 (3)	N1—C17—H16A	109.5
C19—N3—C20	120.5 (3)	C18—C17—H16B	109.5
C20—N5—Na1	171.8 (4)	N1—C17—H16B	109.5
C6—C1—C2	119.6 (4)	H16A—C17—H16B	108.1
C6—C1—C7	119.2 (4)	C17—C18—N2	111.4 (3)
C2—C1—C7	121.1 (3)	C17—C18—H17A	109.4
O2—C2—C1	123.9 (3)	N2—C18—H17A	109.4
O2—C2—C3	117.9 (3)	C17—C18—H17B	109.4
C1—C2—C3	118.2 (3)	N2—C18—H17B	109.4
O4—C3—C4	126.1 (4)	H17A—C18—H17B	108.0
O4—C3—C2	113.4 (3)	N4—C19—N3	173.6 (4)
C4—C3—C2	120.4 (4)	N5—C20—N3	173.8 (4)
C3—C4—C5	120.5 (4)	N1—C16—C9	126.5 (3)
C3—C4—H4	119.7	N1—C16—H20	116.7
C5—C4—H4	119.7	C9—C16—H20	116.7

### Hydrogen-bond geometry (Å, °)

**Table:**

*D*—H···*A*	*D*—H	H···*A*	*D*···*A*	*D*—H···*A*
O5—H5A···N3^i^	0.82	2.14	2.960 (4)	175
O5—H5B···N4^ii^	0.82	2.03	2.852 (4)	177

Symmetry codes: (i) *x*−1, *y*, *z*; (ii) *x*−1, −*y*+1/2, *z*−1/2.
